# Immunization with the pan-fungal vaccine, NXT-2a, reduces fungal burden following serial intravaginal challenges with *C. albicans* in a non-human primate model of experimental vulvovaginal candidiasis

**DOI:** 10.1371/journal.pone.0352577

**Published:** 2026-06-29

**Authors:** Daniel A. Wychrij, Whitney Rabacal, Taylor I. Chapman, Hubertine M.E. Willems, Casey Ellen Pac, Kwadwo O. Oworae, Karen A. Norris

**Affiliations:** Center for Vaccines and Immunology, Department of Infectious Diseases, University of Georgia, Athens, Georgia, United States of America; University of the Witwatersrand, SOUTH AFRICA

## Abstract

Worldwide, recurrent vulvovaginal candidiasis (RVVC) is estimated to affect approximately 138 million women annually. Current treatments for RVVC include azole therapies; however, these frequently fail to prevent recurrence and are limited by potential teratogenic effects and increasing azole resistance. Despite these limitations, there are no approved fungal vaccines. While rodent models have advanced our understanding of disease pathogenesis and vaccine development, their variable susceptibility to human pathogens and limited ability to recapitulate human immune responses restrict translational progress. Here, we established a nonhuman primate model of VVC reinfection in Japanese macaques (*Macaca fuscata*) and used this model to evaluate the immunogenicity and protective efficacy of the ‘pan-fungal’ vaccine candidate NXT-2. We show that like humans, Japanese macaques are susceptible to VVC infections with *C. albicans* and display evidence of active infection through the formation of hyphae and recruitment of polymorphonuclear cells within the vaginal lumen. Infection and reinfection induced limited anti-*Candida* IgG responses and did not prevent reinfection. Notably, immunization of *C. albicans* reinfected macaques with NXT-2 induced robust systemic and mucosal anti-NXT-2 IgG antibody titers and reduced fungal burden in the vaginal lumen following subsequent rechallenge. Together, these data establish a valuable preclinical model for investigating host responses to VVC reinfection and for vaccine development, highlighting the potential of NXT-2 as a vaccine candidate to prevent RVVC.

## Introduction

Vulvovaginal candidiasis (VVC) is an increasingly common fungal infection of the lower female reproductive tract. Most infections are caused by *Candida albicans* (*C. albicans*), but infections due to other *Candida* species are rising in frequency [1]. *C. albicans* is part of the normal vaginal microbiota and commonly colonizes the vaginal lumen asymptomatically [2]; however, during symptomatic infection, fungal overgrowth results in mucosal damage and inflammation of the vaginal epithelium [3]. Common symptoms of infection include localized pain, vaginal discharge, vulvar pruritus, vulval edema and dyspareunia [4]. Approximately 75% of all women will have at least one episode of VVC in their lifetime [5] and 5–8% of women will suffer from recurrent VVC (RVVC), defined as three or more cases per year [6,7]. Unlike invasive fungal infections which predominantly affect immunocompromised individuals [8], VVC and RVVC are infections of immunocompetent women [9]. These infections negatively impact the quality of life of women with (R)VVC and place a large economic burden on society. Current global estimates indicate RVVC affects approximately 138 million women annually and could be responsible for up to $14.39 billion per year in lost productivity [6]. It is estimated that in the US alone, (R)VVC treatment costs exceed $370 million, with lost productivity estimated at $4.7 billion [6,10].

Treatment strategies for VVC are determined by the classification of infection. Uncomplicated VVC infections (infrequent infections with mild or moderate severity primarily caused by *C. albicans*) [4] are often treated with a short course of azoles, with fluconazole being the most frequently prescribed [5,11]. Azole regimens are generally effective against VVC [4], but they do not reliably prevent recurrent infections after treatment is discontinued. Current guidelines for the treatment of RVVC recommend induction azole regimens followed by maintenance therapy for 6 months [4,11]. Extensive and prolonged use of a single class of antifungal agents, such as azoles, has likely contributed to the emergence of azole-resistant *Candida* species [12,13]. In addition, increasing evidence suggests that long-term use of high dose fluconazole may be teratogenic [14,15]. This is especially concerning considering the high frequency of RVVC among women of child-bearing age [16]. The non-azole drug, ibrexafungerp, a triterpenoid, is approved for VVC and RVVC, however, it is contraindicated during pregnancy [17].

Due to concerns over antifungal resistance, the limited ability of azoles to prevent recurrence, and therapeutic limitations of available drugs in women of childbearing age, RVVC susceptible patients would benefit from immunotherapeutic alternatives such as vaccines designed to modulate host responses and to reduce disease burden or recurrence. Several target antigens have been assessed as potential antifungal vaccine candidates [18]. Two vaccine candidates, PEV7 [19] and NDV-3 [20], demonstrated protective effects in rodent models of VVC, supporting their subsequent evaluation in clinical trials [21–24]. PEV7 (NCT01067131) [22] has not progressed beyond Phase 1 trials, while NDV-3 (NCT01926028) was shown to be safe and immunogenic during a Phase 1b/2a trial, and increased the time between recurrence in women under 40 years of age [24]; however, it has not been approved for use.

To address the global need for novel strategies to prevent and treat fungal infections that pose a significant threat to public health, we previously developed the ‘pan-fungal’ vaccine candidate NXT-2. NXT-2 is a 90-amino acid consensus peptide, based on the conserved amino acid sequence of the KEX1 regions from the pathogenic fungi *Candida albicans*, *Aspergillus fumigatus*, *Pneumocystis jirovecii* and *Cryptococcus neoformans* [25]. Immunization with NXT-2 is highly immunogenic and provides broad protection against multiple pathogens causing invasive infections in immunosuppressed murine models of pulmonary aspergillosis, invasive candidiasis due to *C. albicans* and *C. auris*, and in non-human primate (NHP) models of pneumocystis pneumonia [25–27]. To further evaluate the broad protective efficacy of NXT-2 as a pan-fungal vaccine candidate, we evaluated the protection provided against a localized, mucosal infection. We demonstrated that NXT-2 is protective in a murine model of VVC challenged with *C. albicans* [28]. Peripheral immunization with NXT-2 induced a robust increase in systemic antibody titers in the plasma and, notably, in the vaginal mucosa. Following intravaginal challenge with *C. albicans,* NXT-2 immunized mice had reduced vaginal fungal burden, polymorphonuclear (PMN) infiltration, and tissue damage compared to sham immunized controls [28]. Overall, these findings support further preclinical evaluation of this candidate against VVC.

Rodent models have played a critical role in understanding the pathogenesis of VVC and the development of promising vaccine candidates, but they may not accurately predict success in clinical populations. Unlike humans [29–31] and NHPs [32], commercially available rodents are not naturally colonized with *C. albicans* at mucosal sites [33]. NHPs are more closely related to humans than murine models and share similar reproductive tract anatomy, menstrual and hormone cycles [34,35], and immune profiles in the localized reproductive tract [36,37]. NHPs have also been widely used to study vaccine-induced immunity and localized immunity against sexually transmitted diseases in women [38–40]. In addition, NHPs provide a more suitable model system to study repeated exposure to *C. albicans* because their longer lifespan compared to rodents allows for serial sampling within the same animal without substantial aging [41]. Collectively, these features make NHPs a valuable model for studying natural and vaccine-induced mucosal immunity following repeated exposure to *Candida*.

The development of a more clinically relevant model is essential to better predict the success of (R)VVC vaccine candidates prior to clinical trials. To date, only one previous study has been performed to assess the susceptibility of NHPs to VVC infection [32]. In this study conducted by Steele et al., estrogen-treated rhesus macaques (*Macaca mulatta*) were intravaginally challenged with *C. albicans* and fungal burden was assessed. All animals became actively infected, with peak fungal burden observed on day 4. Of note, unlike rodents, macaques were naturally colonized with *C. albicans* at mucosal sites and had prior immune sensitization, similar to humans. Ultimately, this study supported the feasibility of VVC infections in NHPs [32]. However, the susceptibility of NHPs to repeated experimental infection, akin to recurrent disease in humans, has not been previously addressed.

To address this issue, we examined the course of experimental vaginal infection with *C. albicans* in Japanese macaques *(Macaca fuscata)* and established conditions for serial intravaginal infections*,* providing a preliminary model for studying repeated vaginal exposure. It has been speculated that the presence of *Candida spp*. may shape anti-*Candida* immune responses, potentially leading to commensalism-induced immunity in VVC [33]. However, how prior exposure to *Candida* in the vaginal lumen plays a role in immunity against RVVC remains unclear in the context of recurrent disease. We explored the effects of repeated experimental challenge on both the systemic and localized mucosal antibody responses against *C. albicans.* We then used this model to evaluate the immunogenicity and antifungal efficacy of the pan-fungal vaccine candidate, NXT-2, by measuring fungal burden and anti-*Candida* humoral responses following immunization and subsequent experimental rechallenge.

## Results

### Japanese macaques are susceptible to repeated experimental infection with *C. albicans*

**Fig 1:**
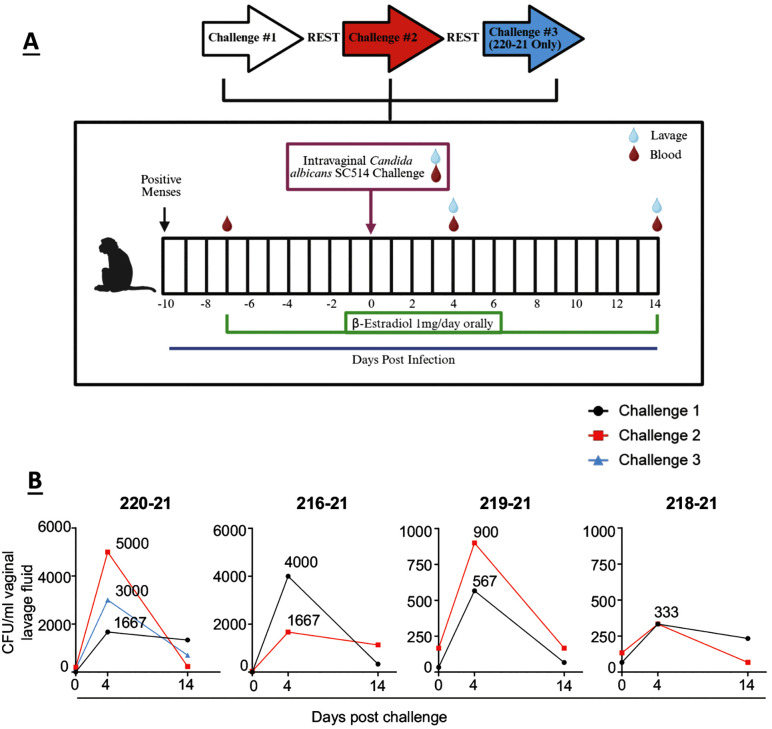
Serial intravaginal *Candida albicans* challenge in Japanese macaques. **A**) Experimental design for serial intravaginal challenge. Japanese macaques were monitored daily for menstruation. Following a positive menstruation swab, animals were rested for 3−4 days before the initiation of oral estradiol administration, which began 7 days prior to intravaginal challenge with *C. albicans* SC5314 and continued for 14 days post-challenge. Blood and vaginal lavage samples were collected prior to challenge and at 4- and 14- days post-challenge. Animals were rested for at least 30 days between challenges before undergoing subsequent intravaginal challenge. One animal (220−21) underwent three serial challenges. **B**) Vaginal burden following serial intravaginal challenge. Vaginal lavage fluid was collected prior to challenge (day 0) and at 4- and 14- days post-challenge, and fungal burden was quantified by plate counting and expressed as CFU/ml. Individual animal data are shown for each challenge.

We sought to evaluate the susceptibility of Japanese macaques to repeated experimental infection with *C. albicans* to generate a reinfection model*.* To assess susceptibility to initial infection, macaques were assessed for menstruation before being subsequently administered oral estradiol 7 days prior to intravaginal challenged with *C. albicans* SC5314 (Fig 1A). Fungal burdens were then assessed in vaginal lavages over a period of 14 days. Following the challenge, the fungal burden peaked at 4 days post-challenge, with all animals having detectable vaginal fungal burdens throughout the 14 days of post-challenge observation (Fig 1B).

Next, we sought to determine if VVC experienced macaques are susceptible to subsequent experimental challenge. Following the initial challenge, estradiol administration was stopped, and the Japanese macaques were rested for a period of at least 30 days. After this rest period, estradiol was administered following menstruation. The Japanese macaques were then challenged again with *C. albicans* and assessed for 14 days (Fig 1A). Following subsequent experimental challenge, vaginal fungal burdens again peaked at 4 days post-challenge and were detected throughout the 14 days. In NHPs 220-21 and 219-21, the fungal burden at 4 days post-challenge during this reinfection was higher than the primary infection (Fig 1B; 220-21 - 5000 CFU/ml vs. 1667 CFU/ml and 219-21 - 900 CFU/ml vs. 567 CFU/ml), while in 218-21, the fungal burden was comparable to the first challenge (Fig 1B; 333 CFU/ml in both infection and subsequent challenge). In NHP 216-21, the fungal burden at day 4 was higher in the initial infection compared to the reinfection; however, the reinfection fungal burden was still 1667 CFU/ml (Fig 1B). As this was a pilot study, we further assessed whether 220-21, which had the highest fungal burden at 4 days post-challenge during the reinfection compared to other animals, could be challenged and infected a third time. During this third challenge, we observed a similar trend, with the vaginal fungal burden peaking at day 4 and *C. albicans* being detected up to 14 days post-challenge. These data indicate that Japanese macaques are susceptible to experimental infection with *C. albicans* and that prior infection does not prevent reinfection in this model.

### Intravaginal challenge and rechallenge with *C. albicans* induce active infection in Japanese macaques

**Fig 2:**
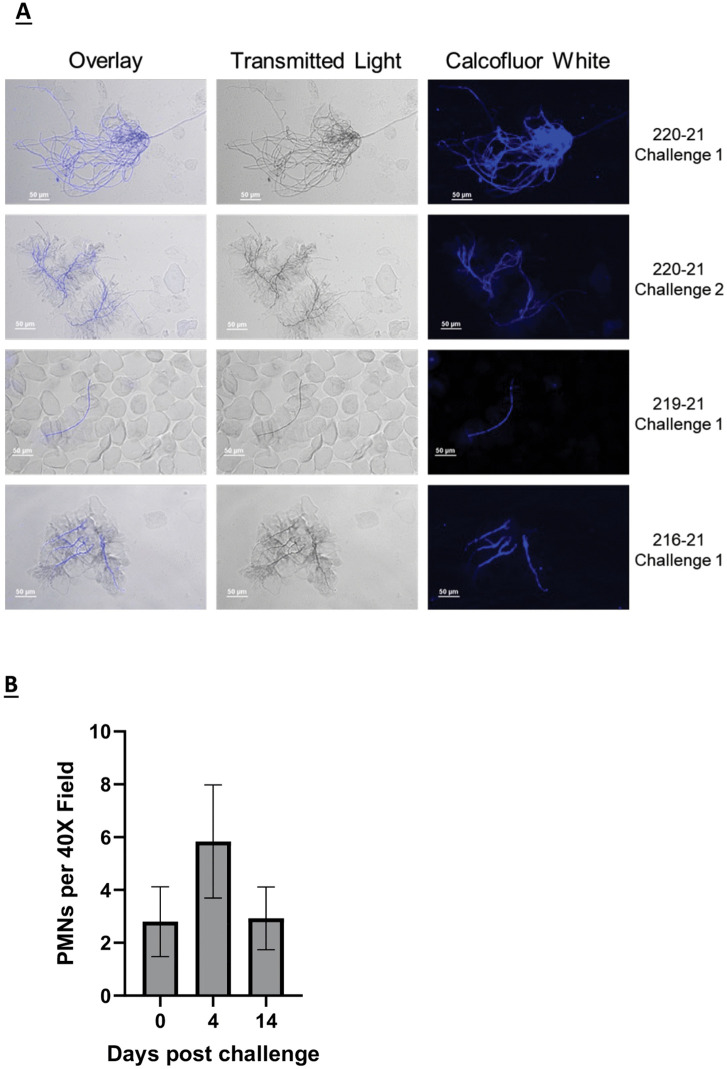
Intravaginal challenge and rechallenge with *C. albicans* induces an active infection in Japanese macaques. **A**) The presence of *C. albicans* hyphae was used to confirm active VVC infection and reinfection. Images depict *C. albicans* hyphae imaged using transmitted light or stained using calcofluor white, as well as the overlayed image. Representative images of hyphae in animals at day 4 post-challenge. Top lane – 220-21, challenge 1. Second lane – 220-21, challenge 2. Third lane – 219-21, challenge 1. Bottom lane – 216-21, challenge 1. Images were taken using the Nikon Ti Eclipse/A1R laser scanning confocal microscope (Nikon Instruments Inc). **B**) Vaginal lavage samples were assessed prior to challenge (day 0) and at 4- and 14- days post-challenge for PMN recruitment by PAP staining. Bars represent the mean ± SEM number of PMNs per 40x field, calculated by averaging 5 non-adjacent fields per sample and combining values across animals from both initial infection and subsequent challenges at each time point post-challenge. Statistical significance was analyzed using Mixed-effects analysis with Sidak’s multiple-comparisons test (0 vs. 4 days post-challenge *P* = 0.1359, 0 vs. 14 days post challenge *P* = 0.9958, 4 vs. 14 days post-challenge *P* = 0.3266).

To determine if intravaginal challenge and subsequent experimental challenge in our model induces active infection, we examined vaginal lavage samples for the presence of *C. albicans* hyphae (Fig 2A) and PMN infiltration (Fig 2B). The presence of hyphae, arising from yeast-to-hypha transitions, is regarded as a marker of active germination and is critical for disease pathogenesis [42]. In addition, in human studies, symptomatic VVC infection is positively correlated with an increase in PMN recruitment [43]. Hyphae were detected by calcofluor white staining of vaginal lavage samples from all animals at the day 4 time point, where fungal burden was at its highest (Fig 2A). When assessing the PMN counts in lavage fluid pooled across animals from both the initial infection and reinfection, we observed an increase at 4 days post-challenge, from 2.80 ± 1.32 to 5.84 ± 2.14 (Fig 2B), which was associated with peak fungal burden. We observed high levels of blood in the lavage of 220−21 during challenge 2 and excluded this data set from analysis. These data provide evidence of active infection and inflammatory responses to infection following intravaginal challenge and rechallenge in our model.

### Localized mucosal *C. albicans* challenge elicits limited anti-*Candida* antibody responses

**Fig 3:**
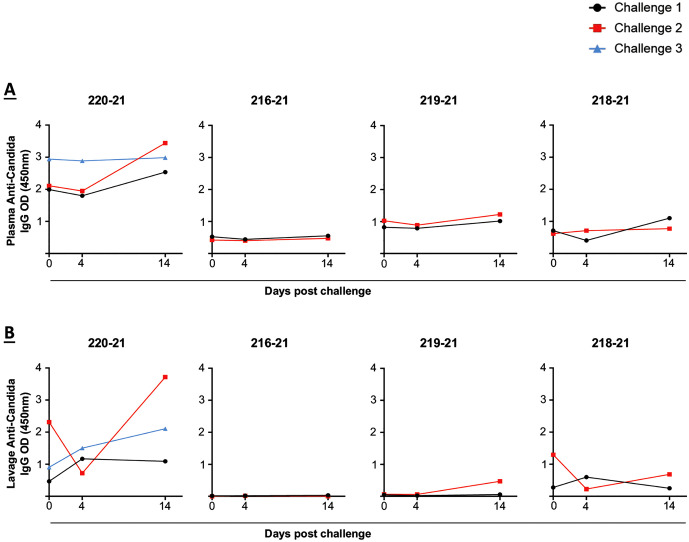
Anti-*Candida* IgG antibody response following infection and reinfection in plasma and vaginal lavage. **A**) Plasma levels of anti-*Candida* IgG assessed by ELISA prior to challenge (day 0) and 4- and 14- days post-challenge. **B**) Vaginal lavage anti-*Candida* IgG levels assessed by ELISA prior to challenge (0) and 4- and 14- days post-challenge.

To assess the host antibody response over the course of initial and subsequent intravaginal challenge with *C. albicans* we evaluated the anti-*Candida* IgG levels in the plasma and vaginal lavage fluid of Japanese macaques. Prior to the intravaginal challenge, all animals had detectable anti-*Candida* IgG plasma titers, demonstrating that these Japanese macaques had prior sensitization to *C. albicans* (Fig 3A). For three of the animals (216-21, 218-21 and 219-21), their anti-*Candida* plasma IgG levels remained consistent over the course of the initial infection. Following subsequent infection, there was no notable increase in these levels regardless of the fungal burdens observed. When compared to the other three animals, 220-21 had higher anti-*Candida* IgG plasma levels (OD value of 2) prior to challenge. During the initial infection and subsequent challenge, there was an increase in anti-*Candida* IgG plasma levels after 4 days (initial infection OD increased from 1.80 to 2.53, reinfection OD increased from 1.95 to 3.44); however, during the third infection, these levels remained consistent at an OD of approximately 3 throughout (Fig 3A).

We further assessed anti-*Candida* IgG responses in vaginal lavage samples as IgG is the major immunoglobulin isotype detected in the female reproductive tract [44,45]. When assessing vaginal anti-*Candida* IgG levels, there was a varied response (Fig 3B). During the reinfection, two of the animals had a slight increase in anti-*Candida* IgG levels after 4 days (218-21; OD 0.23 to 0.68 and 219-21; OD 0.059 to 0.47). Mirroring the anti-*Candida* IgG levels in the plasma, 220-21 had the highest levels in the vaginal lavage through all three infections (Fig 3B). Collectively these data indicate that following infection and reinfection, anti-*Candida* IgG responses are limited and are not associated with enhanced fungal clearance or the prevention of reinfection.

### Vaccination with NXT-2a induces systemic and localized antibody responses in the plasma and vaginal mucosa

**Fig 4:**
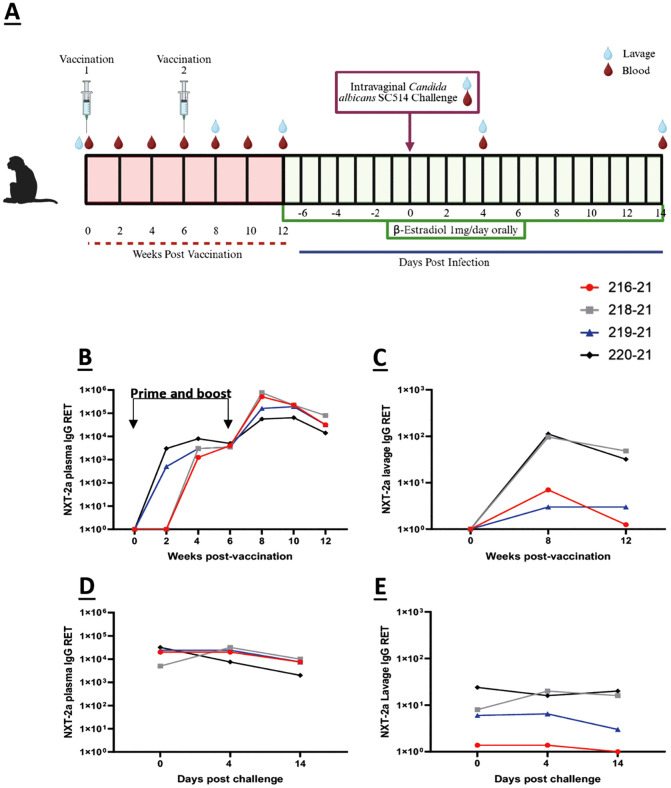
NXT-2a induced IgG responses following immunization and challenge in serially infected Japanese macaques. **A**) Schematic of the vaccination and challenge schedule. Following the establishment of the serial challenge model, the four female Japanese macaques were vaccinated intramuscularly with 100 μg NXT-2a formulated with aluminum hydroxide adjuvant and boosted 6 weeks later. Blood and vaginal lavage samples were collected prior to vaccination and at indicated time points post-vaccination to assess vaccine responses. Estradiol administration was initiated 3-4 days following detection of menstruation and continued for 7 days prior to and 14 days following intravaginal *C. albicans* challenge. Blood and vaginal lavage samples were collected prior to challenge and at 4- and 14- days post-challenge. **B**) Plasma anti-NXT2a IgG reciprocal endpoint titers (RET) were assessed prior to vaccination (day 0) and at 2 weeks-, 4 weeks-, 6 weeks-, 8 weeks-, 10 weeks-, and 12 weeks post-vaccination. **C**) Vaginal lavage anti-NXT2a IgG reciprocal endpoint titers (RET) were assessed prior to vaccination (day 0) and at 8 weeks- and 12 weeks- post-vaccination. **D**) Plasma anti-NXT-2a IgG reciprocal endpoint titers (RET) were evaluated prior to challenge (day 0) and at 4- and 14- days post-challenge. **E**) Anti-NXT2a IgG reciprocal endpoint titers (RET) were evaluated in the vaginal lavage fluid prior to challenge (day 0) and at 4- and 14- days post-challenge.

We have previously demonstrated that in a murine model of VVC peripheral immunization with NXT-2 induced antibody responses detectable in the vaginal mucosa and reduced vaginal fungal burden compared to sham-immunized mice [28]. Based on these results, we sought to evaluate the immunogenicity of an affinity tag free variant of NXT-2, NXT-2a, in *C. albicans* experienced macaques and following subsequent intravaginal challenge with *C. albicans*. A summary of the experimental design is shown in Fig 4A. Following completion of the serial challenge studies in Fig 1A, Japanese macaques were vaccinated with NXT-2a and boosted six weeks later*.* Mean plasma NXT-2a IgG titers peaked at two weeks post-boost, with titers (± SEM) of 374,000 ± 163,605 (Fig 4B). Prior to estradiol administration (12 weeks post-vaccination), mean plasma NXT-2a IgG titers were 39,500 ± 14,150 (Fig 4B). At 13 weeks post-vaccination, animals were intravaginally rechallenged with *C. albicans*. At 4 days post-challenge, we observed mean plasma NXT-2a IgG titers of 20,875 ± 5109. By day 14 post-challenge we observed mean titers of 6750 ± 1689 (Fig 4D). In the vaginal lavage fluid, we observed mean NXT-2a IgG titers of 54.5 ± 28.8 at two weeks post-boost (8 weeks post-vaccination) and 21.1 ± 11.4 prior to estradiol administration (12 weeks post-vaccination) (Fig 4C). Titers remained detectable in the vaginal lavage fluid following challenge, with mean titers of 11.0 ± 4.3 at day 4 and 10.0 ± 4.7 at day 14 post-challenge (Fig 4E). We did not observe an increase in anti-*Candida* IgG levels in the plasma (S1 Fig A) or vaginal lavage fluid (S1 Fig B) associated with vaccination. These data demonstrate that parenteral vaccination with NXT-2a induces both a robust systemic immune response and a localized immune response at the vaginal mucosa.

### Vaccination with NXT-2a significantly reduces fungal burden following intravaginal challenge with *C. albicans* in Japanese macaques

**Fig 5:**
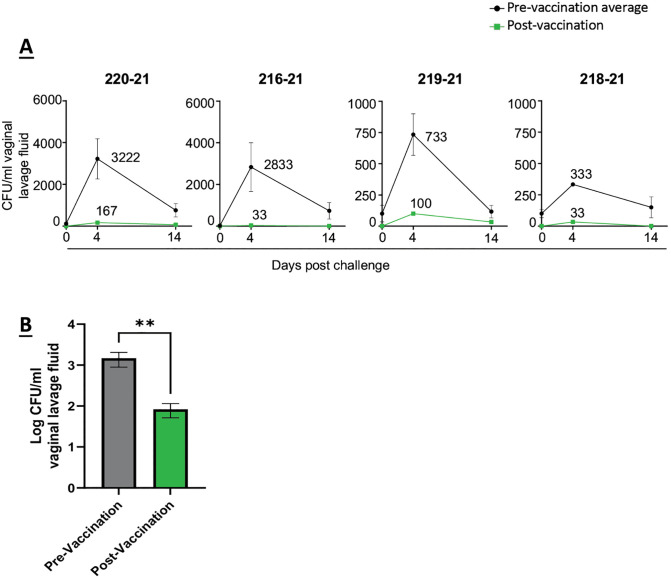
NXT-2a vaccination reduced fungal burden in serially infected Japanese macaques. **A**) Vaginal lavage fluid was assessed prior to challenge (day 0) and at 4- and 14- days post-challenge for fungal burden by plate counting. For each animal, the mean CFU/ml value at each time point from experimental challenges prior to vaccination is presented (black), alongside CFU/ml values from the post-vaccination challenge (green). **B**) Vaccination with NXT-2a significantly reduced the fungal burden at day 4 post-challenge (**P = 0.0055) when compared to day 4 post-challenge values from the most recent pre-vaccination challenge. Data are represented as mean ± SEM. Statistical significance was assessed by using the ratio paired t-test.

To evaluate the antifungal efficacy of NXT-2a vaccination, we compared vaginal fungal burdens following vaccination and challenge to the mean fungal burdens observed during the pre-vaccination challenges. We observed between a 7- to 85- fold reduction in fungal burdens in vaccinated animals (Fig 5A; 220-21 = 19- fold, 216-21 = 85-fold, 219-21 = 7-fold, 218-21 = 10-fold). When comparing day 4 fungal burden during the most recent pre-vaccination challenge to post-vaccination challenge, there was a significant reduction, from log 3.04 to log 1.81 (Fig 5B; *P* = 0.0055).

## Discussion

In this study, we developed a serial experimental infection model of VVC in NHPs as a novel approach to modeling aspects of RVVC. We demonstrate that Japanese macaques intravaginally challenged with *C. albicans* are susceptible to serial experimental infections, which was confirmed by an increase in vaginal *C. albicans* fungal burden, the presence of hyphae, and increased vaginal PMN infiltration following challenge. Importantly, infection and reinfection induce limited anti-*Candida* IgG responses that do not prevent reinfection, similar to RVVC in humans [43]. Intramuscular immunization of serially infected macaques with NXT-2a induced robust NXT-2a-specific IgG titers in the plasma and vaginal mucosa and reduced fungal burden following subsequent challenge. These data support the utility of this model and the efficacy of NXT-2 based immunization strategies against (R)VVC.

We observed a range of peak fungal burdens across the animals following the initial infection (Fig 1B). Some animals had lower fungal burdens than those observed in a previous study conducted by Steele et al. in rhesus macaques [32], but were closer in range to values observed in human studies [43]. Importantly, in the current study, initial infection did not prevent reinfection. Three of the four animals had comparable or higher fungal burdens during this subsequent infection, while the fourth animal had a slightly lower fungal burden; however, the burden remained 1667 CFU/ml. This suggests that following the initial infection, NHPs remained susceptible to subsequent infection. These findings are similar to a clinical study conducted by Fidel et al., which reported that women with an infrequent history of VVC remain more susceptible to symptomatic VVC infection following challenge than those with no history of VVC [43]. Furthermore, in the same study, it was observed that in symptomatically infected individuals, there was a correlation between higher levels of PMNs and higher fungal burden [43]. We observe a similar phenotype in our study, with the average levels of PMNs peaking at 4 days post challenge (Fig 2B) when fungal burden is at its peak (Fig 1B). Together, these findings suggest that the innate immune responses to vaginal *Candida* infections in Japanese macaques share features with those observed in human disease.

Our study demonstrates that Japanese macaques, like humans [46], exhibit prior sensitization to *C. albicans,* a feature that may be important for evaluating vaccine responses and is absent in murine models. All the animals had detectable systemic anti-*Candida* IgG levels prior to experimental *Candida* challenge (Fig 3A). In three of the four animals, intravaginal challenge did not result in increased systemic (Fig 3A) or mucosal (Fig 3B) anti-*Candida* IgG levels following subsequent challenge. One animal (220-21) exhibited elevated systemic and vaginal anti-*Candida* IgG levels; however, this animal also maintained high fungal burdens during challenges. Collectively, these data indicate that naturally acquired anti-*Candida* IgG responses are limited and were not associated with effective fungal clearance or the prevention of subsequent infections in this model.

The limited protective role of infection induced anti-*Candida* antibodies in the Japanese macaque model is consistent with observations in human VVC and RVVC, where anti-*Candida* IgG levels do not differ significantly between symptomatic and asymptomatic individuals or healthy controls [47,48]. While the mechanisms underlying this lack of protection remain unclear, they may involve immunoregulatory mechanisms within the vaginal mucosa or antigen masking that results in non-protective antibody responses. Parallels can be drawn with other mucosal pathogens such as Human Papillomavirus virus (HPV), where natural infection induces weak or undetectable mucosal antibody responses, whereas vaccination elicits robust systemic and mucosal immunity associated with protection [49]. While *Candida* is a commensal organism, these observations support the rationale that vaccination may overcome limitations in anti-*Candida* immunity at mucosal sites.

Here, a prime-boost NXT-2a vaccination regimen elicited robust systemic and localized NXT-2a specific IgG responses that were detectable throughout subsequent *C. albicans* challenge (Fig 4B, C). Vaccination did not increase anti-*Candida* IgG levels systemically or locally (S1 Fig A and B). Although fungal burdens during initial experimental challenges were variable, consistent with heterogeneity seen in human VVC, all animals exhibited active infection prior to vaccination. As a result, each animal served as its own control allowing us to directly compare fungal burden pre- and post-vaccination. Following vaccination, we observed a reduction in fungal burden particularly at day 4 post-challenge (Fig 5A and B). While fungal burden was reduced following vaccination, we still observed a slight peak at day 4 post-challenge. Due to the low fungal burden, we were unable to detect any *C. albicans* hyphae.

The induction of systemic and mucosal NXT-2a specific antibody responses in vaccinated NHPs, together with reduced fungal burden following challenge, supports and extends observations from our murine VVC studies where vaccinated animals had detectable systemic and localized titers and reduced fungal burdens compared to sham-immunized animals [28]. Importantly, the present study evaluates the effects in a NHP serial challenge model that more closely reflects key aspects of human vaginal *Candida* exposure. These results support the conclusion that vaccine-induced immune responses, including antibody-mediated mechanisms, contribute to the modulation of fungal burden following challenge. Moreover, these findings provide further evidence of the broad efficacy of the pan-fungal vaccine NXT-2a. We show that NXT-2a not only provides protection against life-threatening invasive fungal infections in immunosuppressed experimental models, but also against localized, mucosal *C. albicans* infections in immunocompetent models. This is especially significant as VVC and RVVC are associated with high morbidity.

There are limitations to this study. This was an initial study to test the feasibility of NXT-2 immunization against (R)VVC in a more relevant pre-clinical model. Future studies will investigate vaccine dosing, durability, and efficacy in larger cohorts. Given the mucosal nature of VVC, future studies incorporating additional immune parameters, including other antibody isotypes such as IgA, as well as cellular immune responses, will be important for fully defining mechanisms of protection. While this model does not fully recapitulate the clinical complexity of RVVC in humans, the demonstration of susceptibility to experimental reinfections in Japanese macaques provides a reproducible, highly clinically relevant model for evaluating vaccine-associated modulation of vaginal fungal burden following repeated exposure.

In summary, we demonstrate that Japanese macaques are susceptible to experimental vaginal *C. albicans* infection and that this serial intravaginal challenge model can be used to evaluate vaccine-associated effects in NHPs. While naturally acquired anti-*Candida* IgG responses were not associated with protection from subsequent challenge, vaccination with the pan-fungal vaccine candidate, NXT-2a, elicited both robust systemic and localized IgG responses and was associated with a reduction in vaginal fungal burden following rechallenge in previously exposed NHPs. Overall, these findings support the use of a novel, serial challenge NHP model for assessing vaccine-mediated modulation of VVC and provide further rationale for continued preclinical evaluation of NXT-2a as a vaccine candidate against VVC and RVVC.

## Materials and methods

### Animals

Four female Japanese macaques (*Macaca fuscata*) aged 6–7 years were obtained from the Oregon National Primate Research Center (Hillsboro, OR). All studies were approved by the Institutional Animal Care and Use Committee (IACUC) of the University of Georgia. All animal studies were performed in the University Research Animal Resources Facility at the University of Georgia, an American Association for the Accreditation of Laboratory Animal Care (AAALAC)-accredited facility. The University holds Animal Welfare Assurance Number A3437-01. The study was conducted in accordance with the principles and standards set forth in the Principles for Use of Animals (NIH Guide for Grants and Contracts), the Guide for the Care and Use of Laboratory Animals, and the provision of the Animal Welfare Acts (P.L. 89-544 and its amendments). Compliance is validated by the UGA IACUC and regular inspections by USDA veterinarians.

Animals were socially housed (paired) in Allentown primate housing on a 12-hour light-dark cycle in a climate-controlled facility. Animals were fed twice daily with primate chow (Monkey Diet 5038, LabDiet) and fresh fruit and vegetables. Water was provided *ad libitum*. A variety of enrichment strategies were employed including food based and non-food related as recommended by the Guide for the Care and Use of Laboratory Animals. Animals were monitored twice daily by animal care staff to check for health and welfare. All efforts were made to minimize any pain during procedures, with animals anesthetized during sample collection and *C. albicans* challenge. Following the completion of the study animals were used in another study.

### Culture and harvest of *C. albicans*

*C. albicans* strain SC5314 (ATCC) from glycerol stock stored at −80°C was spread onto a yeast extract peptone dextrose (YPD) agar plate and incubated for 48 hours at 30°C. A single isolated colony was selected and cultured in 10 ml of YPD broth at 30°C for 18 hours, shaking at 225 rpm. Stationary phase *C. albicans* cells were washed three times with sterile 1x phosphate buffered saline (PBS) and counted on a hemocytometer.

### Primate model of *C. albicans* vaginitis infection and serial intravaginal challenge

To assess menstruation status, NHPs were swabbed daily at the vaginal introitus, and the presence or absence of blood was recorded. Visible blood was used to confirm menstruation. Following confirmation of menstruation status, animals were rested for a period of 3–4 days. It has been previously demonstrated in Steele et al., that the administration of exogenous estradiol enhanced the susceptibility of rhesus macaques to VVC infection [32]. As a result, animals were administered 1 mg of estradiol (American Health Service Sales Corp) orally. Estradiol administration began 7 days prior to intravaginal inoculation with *C. albicans* and continued daily until 14 days post-challenge.

Seven days after the administration of estradiol, peripheral blood and vaginal lavage fluid samples were collected prior to *C. albicans* challenge. Macaques were intravaginally inoculated with *C. albicans* blastoconidia (5 × 10^6^ − 1 × 10^7^ total) in a volume of 1 mL PBS. To minimize the loss of challenge inoculum, the caudal end of the animal was elevated for 15 minutes, and the macaques were monitored for leakage over a 30-minute period. On days 4 and 14 post-challenge vaginal lavages and blood collection was conducted. Lavage fluid was assessed for hyphal morphology and fungal burden. Daily estradiol administration was stopped at 14-days post challenge and macaques were rested for a period of at least 30 days before undergoing subsequent challenge as described above. Three animals (216-21, 218-21, 219-21) underwent two experimental challenges, while one (220-21) underwent three experimental challenges.

### Sample collection

For blood collection, vaginal lavage and *Candida* inoculation, macaques were anesthetized using ketamine hydrochloride. Blood was drawn from the femoral vein. No more than 10% of the circulating blood volume was drawn in a two-week period. Blood was centrifuged at 3,000 x g for 5 minutes at room temperature and plasma was stored at −80 °C until use. Macaques were vaginally lavaged using 4 ml of sterile 1x PBS using a sterile transfer pipette.

### Fungal burden

Fungal burden was assessed as described in our previous study [28]. Briefly, vaginal lavage fluid was serially diluted 10-fold in sterile 1x PBS and 10 μl was plated in triplicate onto YPD agar plates containing 40 μg/ml chloramphenicol to minimize bacterial outgrowth. Plates were incubated at 37°C for 48 hours and colonies were enumerated. Colony forming units (CFU) per ml (CFU/ml) values were reported.

### PMN infiltration

PMN infiltration was quantified as previously described [28]. Ten microliters of vaginal lavage fluid were spread onto Tissue Path Superfrost Plus Gold slides (Fisher Scientific) and allowed to air dry at room temperature before being fixed with CytoPrep spray fixation (Electron Microscopy Sciences). Slides were stained using the Papanicolaou method (PAP smear) [50]. Under blinded conditions, PMNs were enumerated in 5 non-adjacent fields using light microscopy at 40x objective and mean values were calculated as the average PMN count across 5 non-adjacent fields per sample.

### Assessment of hyphal formation

Following sample processing for fungal burden and PMN assessment, vaginal lavage fluid was centrifuged at 800 x g for 5 minutes. The supernatant was frozen at −80 °C until use and the pellet was collected and diluted 1:100 in sterile 1x PBS. Ten microliters of the diluted pellet sample were added to a standard glass slide followed by 10 μl of calcofluor white stain (Sigma-Aldrich) and 10 μl of 10% potassium hydroxide solution (Sigma-Aldrich) diluted in sterile water. The slides were mounted and visualized under UV light (λEx = 355 nm) using the Nikon Ti Eclipse/A1R laser scanning confocal microscope (Nikon Instruments Inc.).

### Anti-*Candida* ELISA

Anti-*Candida* IgG ELISA assays were conducted using the *C. albicans* IgG ELISA kit (DRG International, Inc.) following the manufacturer’s protocols with the following modifications. Briefly, plasma samples were heat-inactivated and diluted 1:250 in the sample diluent provided, while heat-inactivated vaginal lavage samples were used undiluted. One hundred microliters of neat lavage fluid or diluted plasma was plated in duplicate onto the provided pre-coated plates alongside 100 μl/well of the positive and negative controls provided in the kit. The plates were covered with the provided foil and incubated for 1 hour at 37 °C before being washed 5 times with wash solution. Following washing, 100 μl/well of goat anti-monkey IgG-HRP secondary antibody (Nordic Immunology), diluted 1:10,000 in 5% non-fat milk in sterile 1x PBS (blocking buffer), was added, and the plates were incubated at room temperature for 30 minutes. Plates were washed 5 times with wash solution, and 100 μl/well of provided substrate solution was added before being incubated for 15 minutes at room temperature in the dark. The reaction was stopped following the addition of 100 μl/well of the provided stop solution, and the absorbance was measured at 450 nm and 620 nm. Corrected OD values (OD450 minus OD620) were reported.

### NXT-2a vaccination and *C. albicans* challenge of primates

Following the establishment of a serial experimental challenge model, the four NHPs were rested for a period of at least 3 months prior to vaccination. For these vaccination studies, we used an affinity tag-free variant of the pan-fungal antigen NXT-2, NXT-2a, first described by Rayens et al [25]. NXT-2a was recombinantly expressed and purified as previously described [26]. SDS-PAGE images of the purified NXT-2a are shown in (S2 Fig). Prior to vaccination, NXT-2a preparations were endotoxin-tested using the Pierce Chromogenic Endotoxin Kit (ThermoFisher). Animals were intramuscularly vaccinated with 100 μg of recombinant NXT-2a and Alhydrogel adjuvant 2% (InvivoGen, 0.5 mg Al3+) in a total volume of 500 μl. Animals were boosted with 100 μg of NXT-2a + Alhydrogel adjuvant 2% 6 weeks after the initial vaccination. Peripheral blood samples were collected prior to vaccination (0), and 2-, 4-, 6-, 8-, and 10 weeks- post-vaccination to assess vaccine responses. Vaginal lavages were conducted prior to vaccination (0) and at 8 weeks post-vaccination. Approximately 4 weeks after the initial vaccination, daily monitoring for menstruation began, and continued until two menstruation cycles had been detected. Following detection of the second positive menstrual cycle, NHPs were rested for a period of 3–5 days before the administration of 1 mg of oral estradiol. Estradiol administration commenced approximately 12 weeks after the initial vaccination (6 weeks post-boost) and was administered daily until 14 days post-challenge. Seven days after estradiol administration commenced, peripheral blood and vaginal lavage samples were collected before the animals were intravaginally challenged with *C. albicans* as described above.

### Anti-NXT-2a ELISA

Anti-NXT-2a antibody titers were monitored by ELISA. ELISA assays were conducted using microtiter plates (Immulon 4HBX; Thermo Fisher Scientific) coated with 5 μg/ml of purified NXT-2a in 1x PBS as previously described [26]. Plasma and lavage samples were heat-inactivated at 56 °C for 30 minutes prior to dilution. Plasma samples were initially diluted 1:1000 in 5% non-fat milk in sterile 1x PBS (blocking buffer), and 1:2 serial dilutions were made. Vaginal lavage samples were plated neat (undiluted), followed by 1:2 serial dilutions in blocking buffer. A total of 50 μl/well of sample (neat or diluted) was added to the NXT-2a-coated plate. All samples were run in duplicate. Plates were incubated at 4 °C overnight and then washed four times with PBS-T (0.05% Tween-20). Following washing, 100 μl/well of goat anti-monkey IgG-HRP secondary antibody (Nordic Immunology), diluted 1:10,000 in blocking buffer, was added, and plates were incubated at 37 °C for 1 hour. Plates were subsequently washed six times with PBS-T and 100 μl/well of TMB (BD Biosciences) was added to develop the plates. The reaction was stopped with 50 μl/well of 1 M sulphuric acid and absorbance was measured at 450 nm. All assay plates had internal controls. Archival samples from control animals with no detectable titers were used as negative controls, while archival samples from vaccinated animals with known titers were used as positive controls.

### Statistical analysis

Data were plotted and statistically analyzed using GraphPad Prism (GraphPad Software, Version 11.0.0 (84)). Fungal burden prior to the last challenge (pre-vaccination) and post-vaccination was analyzed using a ratio paired two-tailed t-test. PMN infiltration during challenge and rechallenge was analyzed using mixed-effects analysis with Sidak’s multiple-comparison test. Data are reported as mean ± standard error of the mean (SEM).

## Supporting information

S1 Fig Anti-*Candida* IgG antibody responses following NXT-2 immunization and challenge of serially infected Japanese macaques. **A**) Plasma anti-*Candida* IgG levels were assessed by ELISA following vaccination and subsequent *C. albicans* challenge (green) at day 0 (prior to challenge) and at 4- and 14- days post-challenge. For comparison, mean anti-*Candida* IgG plasma levels from experimental challenges prior to vaccination are shown (black). **B**) Vaginal lavage anti-*Candida* IgG levels were assessed by ELISA following vaccination and subsequent *C. albicans* challenge (green) at day 0 (prior to challenge) and at 4- and 14- days post-challenge. Mean anti-*Candida* IgG levels in the vaginal lavage from experimental challenges prior to vaccination are shown for comparison (black).
(TIF)

S2 Fig Purified NXT-2a. Purified NXT-2a was run on an SDS-PAGE gel and stained with Coomassie Blue (left) and analyzed by Western blot (right). M, molecular weight standard; arrow points to expected protein size (~10kDa).
(TIF)

S1 Data Fungal burden values. (XLSX)

S2 Data PMN values. (XLSX)

S3 Data anti-Candida IgG Plasma and lavage OD values. (XLSX)

S4 Data RET values after vaccination and challenge. (XLSX)

S5 Data Fungal burden pre and post vaccination. (XLSX)

S6 Data anti-candida IgG plasma and lavage pre&post vax. (XLSX)
